# Chilean gender identity support program in children and adolescents: review of a public health policy

**DOI:** 10.3389/fpubh.2026.1832067

**Published:** 2026-05-14

**Authors:** Claudio Martinez, Isidora Paiva, Sofia Salas

**Affiliations:** 1 Universidad Diego Portales, Psychology Faculty, Santiago, Chile; 2 Universidad Adolfo Ibanez Escuela de Psicologia, Peñalolén, Chile; 3 Universidad del Desarrollo Facultad de Medicina, Santiago, Chile

**Keywords:** gender identity, gender identity in children and youth, gender identity policies, gender identity programs, health services for transgender persons

## Abstract

Trans and gender non-conforming (TGNC) children and adolescents face a disproportionately high risk of mental health difficulties, including depression, anxiety, and suicidal behaviors. In recent years, their care and protection have become the focus of intense political and societal debate, particularly regarding the evidence base for psychological and medical interventions. Within this polarized climate, TGNC youth and their families are often caught between conflicting perspectives, while their need for emotional support, social recognition, and reliable evidence-based information remains both urgent and legitimate. These challenges are embedded in structural inequities rooted in historical, legal, and cultural systems, which call for concrete state responses grounded in social justice and human rights. This article examines the Gender Identity Support Program (PAIG in Spanish) in Chile, implemented nationally since 2022, as a pioneering public policy initiative explicitly designed to support TGNC children, adolescents, and their families. The paper describes the legal and institutional framework underpinning the program, its guiding principles, core intervention strategies, and the main challenges encountered during its implementation. In addition, it discusses the program’s strengths, as well as structural and political vulnerabilities that may threaten its long-term sustainability. By presenting PAIG as a case study, this article contributes to the international discussion on how public policies can effectively address the mental health needs and social inequities affecting TGNC youth, highlighting the role of state-level interventions in promoting inclusion, well-being, and equity for gender-diverse populations.

## 1 Introduction

The term *transgender*,^1^ or simply *trans*, is an inclusive concept encompassing a diverse group of individuals who experience an incongruence between their gender identity and the gender assigned to them at birth based on natal sex (1, 2). This experience of incongruence may emerge early in development through bodily discomfort or through aspects related to social expression (e.g., clothing, gender presentation, etc.). For this reason, the term *trans* or *transgender and gender non-conforming* (TGNC) includes both individuals who explicitly identify as transgender and those who, at that stage, express discomfort with their gender assigned at birth (3, 4).

Globally, research estimates that individuals who identify as transgender represent between 0.3 and 0.5% of the adult population and between 1.2 and 2.7% of children and adolescents. When the broader concept of *gender diversity* is used, these estimates increase to between 2.5 and 8.4% among adults and children/adolescents, respectively (5).

Over the past decade, there has been a sustained increase in the number of children and adolescents expressing incongruence between the sex assigned to them at birth and their experienced gender identity (6, 7). This trend has been documented by specialized clinical services in Europe, North America, and Oceania, which report a significant increase in referrals in recent years for cases of gender dysphoria among adolescents or gender incongruence^2^ in the case of children (8).

In Chile, results from the latest national health survey on Sexuality and Gender (ENSSEX), conducted between 2022 and 2023 with participation from 20,392 individuals, indicate that at least 1% of the population aged 18 years and older identifies as transgender or non-binary (9). Population-based data for children and adolescents are not yet available; however, a study conducted by Organizando Trans Diversidades (OTD) in Chile with a sample of 315 transgender individuals found that 50.1% reported recognizing their gender incongruence between ages 0 and 18, and 7.6% before age 12 (10).

Transgender children and adolescents face an elevated risk of mental health problems, as numerous studies have shown that this marginalized population presents higher rates of depression, anxiety, and suicidal behaviors (11, 12). In Chile, the OTD study found that 56% of respondents reported at least one suicide attempt, with 48% of these occurring between the ages of 11 and 15 (10). Therefore, early access to supportive interventions is essential to promote the well-being and safety of TGNC individuals, particularly children and adolescents (3).

Gender-diverse identities are not pathological and are no longer classified as mental disorders (13). However, the incongruence between a person’s gender identity (their internal sense of gender) and their external physical characteristics may generate psychological distress, often caused by external factors such as stigma and discrimination within social environments, including families, schools, and society more broadly (14, 15). Experiences of gender discordance during childhood and adolescence, therefore, require forms of support that address both the individual distress they may provoke and the tensions that often emerge within family and social contexts.

The design and implementation of support programs for TGNC individuals have become the subject of debate in many parts of the world, often resulting in increased uncertainty and distress for young people and their families. Nevertheless, providing such programs constitutes a responsibility of states and societies in fulfilling the human right to the protection of children (16).

This article describes the *Gender Identity Support Program* (Programa de Apoyo a la Identidad de Género, PAIG in Spanish) in Chile, implemented in 2022, as a pioneering public policy initiative aimed directly at TGNC children and adolescents and their families. As a state-funded program operating nationwide under the supervision of the Chilean Ministry of Health, it currently represents a unique case in Latin America and globally due to its design and scope, although it is not exempt from the tensions arising from broader social and political contexts.

## 2 Mental health of TGNC children and adolescents

The Minority Stress Model (17) posits that social conditions creating chronically hostile and stressful environments can lead to elevated levels of psychological distress and poorer mental and physical health outcomes among minority populations. These structural factors may increase subjectively processed and internally experienced stress, such as stigma or internalized transphobia (18), expectations of rejection, and the need to conceal one’s identity. Together, these stressors can negatively affect the mental and physical health of TGNC individuals, increasing the risk of depression, suicidal ideation, and substance use, among other outcomes (17, 19). Chilean data in adult TGNC individuals confirm a high frequency of depression, suicide attempts, and high levels of anxiety, in accordance with the existing literature (20).

This model also highlights the role of external supports in mitigating the harmful effects of these stressors and promoting positive mental health outcomes. In particular, feelings of belonging to a community and being supported by significant others are associated with higher levels of life satisfaction and meaning in life (21). These experiences contribute to the development of authenticity, pride, and improved self-esteem, which in turn enhance well-being and mental health (22). Community support for TGNC youth, therefore, provides an important scaffolding that offers safe spaces, access to resources, and opportunities to connect with peers. However, the availability of such resources varies widely across communities and is often shaped by demographic factors, economic conditions, and political climates (23).

Because childhood and adolescence are critical stages of psychological and social development, society—and particularly health systems—has the responsibility to safeguard the well-being of these young people by ensuring that care is ethical, prudent, and evidence-based (14, 15).

Medical and psychosocial practices aimed at supporting TGNC children and adolescents have become the focus of intense controversies involving clinical, political, and moral questions, including debates surrounding identity, autonomy, bodily integrity, sexuality, and human rights (24–26). Recent controversies surrounding medical treatments for minors extend far beyond scientific debate and are embedded in broader cultural and political dynamics (27).

For some, early access to gender-affirming interventions represents an issue of civil rights and psychological well-being (15, 23), while for others it remains an area marked by clinical uncertainty that warrants critical examination of the available evidence (26).

One reason this field remains controversial is that diagnoses of gender dysphoria or gender incongruence are based largely on self-perception and behavioral expressions, without any biological marker or laboratory test to confirm them (28, 29). Moreover, these expressions may fluctuate throughout adolescence, a developmental period characterized by identity exploration and emotional variability. During this stage, young people transition from dependence on caregivers toward adult independence, while ongoing changes in the body, brain, and behavior interact continuously with the surrounding environment—home, school, and community—to shape mental health trajectories into adulthood (30).

Gender norms and stereotypes may also increasingly influence mental health during adolescence and affect how mental health conditions are diagnosed and treated (16, 31). These complexities require careful clinical consideration and the involvement of interdisciplinary teams capable of addressing the psychological and family dimensions of each case (32).

Within this context of tension and debate, families and young people often find themselves navigating conflicting perspectives. Their need for emotional support, social understanding, and reliable medical information is both urgent and legitimate. Ensuring that clinical and family decisions are grounded in scientific evidence, psychosocial support, and ethically informed deliberation is therefore essential to protect their well-being and autonomy (14, 15).

Moreover, psychological, social, and health care practices—particularly when directed toward children and adolescents—are never value-neutral; they are intrinsically linked to moral orders and to the recognition of human vulnerability (33). Questions surrounding gender thus become a contested terrain where the boundaries between biological sex and socially constructed gender are negotiated. Diverging moral and political interests often shape the logic of care, framing autonomy not as a purely individual matter but as one mediated by institutional values and ethical considerations (34).

Contemporary cultural and political debates about the rights and lives of TGNC people have direct implications for how health care is conceptualized and organized. Clinicians frequently report uncertainty when confronted with disagreements about appropriate standards of care, the strength of empirical evidence, and the conditions under which informed consent should be obtained (35). However, as Lynch et al. (36) note, the moral and emotional dilemmas inherent in caregiving are not unique to gender identity but are present across many areas of health care. Recognizing this broader context helps counter narratives that frame transgender health care as an exceptional or isolated domain.

For individuals referred to gender identity clinics or support programs, the uncertainties and controversies surrounding care can translate into precarious access to services. At the same time, reducing care to rigid standards of effectiveness or singular moral frameworks becomes increasingly untenable (37). Instead, attention should focus on the broader consequences of care—its social, emotional, and ethical impacts—and its role in everyday life.

In environments characterized by limited resources and discrimination, the effects on TGNC children and adolescents and their families may be particularly harmful. Increasing uncertainty regarding the protection of rights and access to health care has been associated with heightened anxiety and depressive symptoms, particularly in contexts where anti-trans policies have been implemented (38). Campaigns supporting discriminatory laws targeting sexual and gender minorities can lead to increased harassment, heightened stigma, reduced perceptions of safety, and diminished access to health care (39, 40). Consequently, rather than restricting care for TGNC youth, states have a responsibility to implement policies and programs that support and protect their mental health and well-being (16).

## 3 Psychosocial support programs for TGNC children and adolescents

Psychosocial programs for TGNC youth aim to provide mental health support, promote well-being, and assist young people in navigating their gender identity and expression. These programs commonly include individual and family therapy, peer support groups, and connections with community networks. Their goals are to address gender dysphoria, reduce mental health symptoms, and foster a sense of belonging and resilience.

Such programs offer safe environments where transgender youth can explore their gender identity, address mental health concerns through affirming approaches, and develop more effective coping strategies for navigating discriminatory environments (23, 41). They also support families in understanding and accompanying their children’s experiences of gender incongruence, helping to resolve conflicts and build supportive networks. In many cases, these programs encourage families to become advocates for TGNC individuals and to foster more inclusive environments.

Additionally, they facilitate connections between young people and relevant organizations, support services, and health care providers. Peer interaction is another important component, as it provides opportunities for TGNC children and adolescents to share experiences and build a sense of community.

Importantly, these programs must also recognize that gender-nonconforming youth may face distinct challenges linked to other dimensions of identity, such as race, ethnicity, or socioeconomic status. These intersecting factors can either enhance or hinder access to support and resources (41).

In recent years, several countries have developed public or institutional programs acknowledging the need for families to receive guidance and support when their children express gender incongruence. Most of these initiatives operate at local or regional levels, and only a small number provide nationwide coverage.

Table 1 summarizes public or institutional psychosocial programs that serve transgender and gender-diverse children, adolescents, and their families.

**Table 1:** International public psychosocial support programs for transgender youth and families.

Country / Region	Program / Service name	Institutional / Public nature	Target age group / Recipients	Coverage
England (UK)* (66)	NHS Children and Young People’s Gender Service—London Hub	National Health Service (public)	Children and young people up to 18 years; family involvement	National (England) via regional hubs
England (UK)* (67)	NHS Children and Young People’s Gender Service—Northwest Hub	National Health Service (public)	Children and young people up to 18 years; family involvement	Regional (Northwest England)
Australia (68) (Victoria)**	RCH Gender Service—Royal Children’s Hospital (Melbourne)	Statewide public hospital service	Children and adolescents (up to ~16 years) and caregivers	Statewide (Victoria)
Australia (69) (Queensland)**	Queensland Children’s Gender Service	Statewide public hospital service	Children and young people with gender diversity and their families	Statewide (Queensland)
Australia (70) (Western Australia) **	Gender Diversity Service—Perth Children’s Hospital	Statewide public health service	Children and adolescents (up to 18 years) and families	Statewide (Western Australia)
United States (71) (Washington State) ***	Seattle Children’s Gender Clinic	Public pediatric hospital	Adolescents and their families	Local / Regional
United States (Washington, D. C.) *** (72)	Children’s National Gender Development Program	Public academic hospital	Infants, children, adolescents, and families	Local / Regional
United States (Illinois)*** (73)	Lurie Children’s Gender Development Program (Chicago)	Public / academic children’s hospital	Children, adolescents, and families	Local / Regional
United States (74) (Virginia)***	UVA Health Transgender Youth Health Services	Public / academic health system	Youth aged 11–25 and their families	Local / Regional
Netherlands**** (42)	Amsterdam UMC Gender Identity Clinic	University medical center (public)	Children, adolescents, and families	National (Netherlands)
Sweden**** (43)	Gender Dysphoria Team—Karolinska University Hospital	Public / university hospital	Children and adolescents	National (Sweden)

All listed services are publicly funded or integrated into national or state health systems. Most combine psychosocial support, family counseling, and medical care. *The UK is restructuring its national system following the Cass Review Recommendations (75). **In Australia, coverage is typically statewide rather than national. ***U. S. programs are hospital-based and generally have local or regional reach. ****The Netherlands and Sweden maintain national reference centers combining psychosocial, psychiatric, and medical care for gender-diverse youth.

Table 1 shows that, among the few countries that have established public programs, only the Netherlands (42) and Sweden (43) operate with nationwide coverage. In both cases, however, services are centralized in a single institution—typically a university-affiliated center—located in one city. In the United Kingdom, which also provides national coverage, the program that was previously centralized in London has recently been restructured into regional hubs, although it remains under the supervision of the National Health Service (NHS) (44). By contrast, in Chile the PAIG was designed from its inception to offer nationwide coverage and is fully funded and overseen by the Chilean state.

## 4 Gender identity support program (PAIG) in Chile

### 4.1 Legal and regulatory context in which the PAIG emerged

The PAIG program operates within a broad legal and constitutional framework grounded in national and international norms in force in Chile. First, its foundation lies in the recognition and protection of human rights, particularly the rights of children and adolescents, as established in international treaties ratified by the Chilean State and fully incorporated into the domestic legal system, since the 1990 Convention on the Rights of the Child is one of the main, along with others such as the American Convention on Human Rights, known as the “Pact of San José, Costa Rica” from 1990 as well; and the International Covenant on Civil and Political Rights adopted by the General Assembly of the United Nations in 1989. These instruments constitute the normative framework guiding public policies directed at childhood and adolescence from a rights-based perspective.

Within this context, Law No. 20.379 (45) created the Intersectoral Social Protection System and, within it, the comprehensive child protection subsystem *Chile Crece Contigo* [“Chile Grows with You”]. This system aims to support the development of children served within the public health system from pregnancy through 18 years of age, as well as those enrolled in public educational institutions. Its approach is comprehensive, addressing the multiple dimensions that influence child development and coordinating services across areas such as mental health, family dynamics, education, housing, judicial protection, and violence prevention, through collaboration among public and private institutions, under principles of rights protection and respectful parenting.

This framework was further strengthened with the enactment of Law No. 21.430 (46), which explicitly recognizes children and adolescents as full rights-holders, establishes the principle of the best interests of the child as the guiding criterion for decisions affecting them, and guarantees equality and non-discrimination in the exercise of their rights. The law also reinforces the protective role of the family as a fundamental unit of society and recognizes the State’s duty to protect and strengthen it by ensuring policies and programs that provide parents and caregivers with the tools necessary to support the care, education, and well-being of their children.

At the same time, after more than 5 years of legislative debate, Law No. 21.120 (47)—known as the Gender Identity Law—was enacted, marking a historic milestone in the recognition and protection of the rights of transgender people in Chile (48). This law regulates procedures for the rectification of birth records with respect to legal sex and registered name for individuals aged 18 and older, representing a paradigm shift in the recognition of the right to identity. The law is also grounded in guiding principles such as depathologization, dignity in treatment, confidentiality, non-arbitrary discrimination, and respect for progressive autonomy, while also incorporating the best interests of children and adolescents (48). In addition, the Chilean Ministry of Education created a normative allowing students aged 14 to 18 to be addressed by their social name within educational institutions, even when their parents do not agree (49).

Although Law No. 21.120 (47) does not allow administrative rectification of legal sex and name for individuals under 18 years of age, it establishes the State’s obligation to guarantee professional support programs aimed at assisting their development. To this end, the law mandates the Ministry of Social Development and Family (MIDESO), in coordination with the Ministry of Health (MINSAL), to develop regulations ensuring multidisciplinary guidance consistent with a rights-based approach. Within this framework, the health sector plays a central role in safeguarding these guarantees, recognizing gender as a social determinant of health and promoting actions aimed at reducing barriers to comprehensive well-being.

Based on this normative framework, in 2021, the Ministry of Health issued the document *Recommendations for the Implementation of Support Programs for Transgender and Gender Non-Conforming Children and Adolescents*, which provides guidance to teams within the public health system for implementing psychosocial support related to gender identity and establishes the foundations for the creation of the PAIG in Chile (50).

### 4.2 Scope and foundations of the PAIG

The implementation of the PAIG follows the guiding principles established in the Gender Identity Law described above. Operationally, the program consists of multidisciplinary professional guidance that includes psychosocial counseling activities carried out by at least two professionals—typically one psychologist and one social worker—who are hired through public competitive processes within the public health system and are fully funded by the Chilean State.

Its explicit objective is to provide tools that enable the comprehensive development of children and adolescents aged 3 to 17 and their families whose experienced identity and/or gender expression does not correspond to their legal sex and registered name. To achieve this, the program seeks to recognize and validate individual experiences and accompany each person’s process of exploration, respecting their timing, decisions, and subjectivities within a safe and protective environment characterized by dignified and respectful treatment, regardless of how their identity or gender expression is ultimately defined (48).

To fulfill these objectives, the PAIG adopts an intersectoral approach that requires coordination among multiple state institutions at both central and territorial levels, involving three ministries: the Ministry of Health, the Ministry of Education, and the Ministry of Social Development. The program also recognizes the fundamental role of families, who often require guidance and support throughout the process (48).

The program consists of three components: individual psychosocial care, family guidance, and the inclusion of children and adolescents in educational settings, promoting connections with public service networks that respect diverse gender identities and expressions. It also seeks to foster positive and protective experiences throughout the life course, strengthening the ability of young people to cope with potential adverse experiences (48).

Importantly, the PAIG explicitly does not aim to induce or guide participants toward any predetermined identity outcome, nor does it provide hormone therapies. The program operates within specialized outpatient centers in hospitals across the country and may also be delivered by nonprofit organizations^3^ accredited by the Secretariat for Childhood within the Ministry of Social Development and Family. Although the program follows a similar structure and guiding principles in all locations, practical organization, infrastructure, and resources may vary among institutions.

There are multiple pathways for entry into the PAIG: self-referral by the child or adolescent or their family, referral from the National System for the Protection and Guarantee of Children’s Rights, referrals from schools, municipalities, the judicial system, or health care teams (48). All entry pathways require the voluntary participation of the child or adolescent, documented through a formal declaration of willingness; at least one legal guardian must also be informed. If family members initially oppose participation in the program, efforts are made to raise awareness and engage them; if necessary, judicial protection mechanisms may be activated to safeguard the child’s rights.

The program also encourages the participation of at least one adult family member in the support process. However, if family participation is not possible, access to the program is not denied, and the child protection system may assume a supportive role to ensure the protection of the young person’s rights (48).

A fundamental aspect of the program’s design is the strict observance of the right of children and adolescents to be informed and to consent to health care interventions. Based on Law No. 20.584 on Patients’ Rights and Duties in Health Care (51), the program establishes that, before any procedure or treatment, patients must provide informed consent after receiving and understanding all relevant information. Specifically, the program states:

*“Without prejudice to the authority of parents or legal guardians to grant consent in health matters on behalf of competent minors, every child and adolescent has the right to be heard regarding the treatments they receive and to choose among the available alternatives, insofar as the situation allows, taking into account their age, maturity, mental development, and emotional and psychological state. It must be recorded that the child or adolescent has been informed and heard.”* (48).

Once enrolled in the program, the support process includes three phases:

1. **Entry and comprehensive assessment**, involving psychosocial evaluations at the individual, family, and educational levels. This phase provides initial guidance and leads to the development of a care plan.
2. **Implementation of the care plan** at the individual, family, and educational levels, which may include psychological support, family outreach, and coordination with schools.
3. **Program completion**, involving a final assessment of risk and protective factors, determination of continuation, re-entry, or referral, and the preparation of a participation report.

These phases—whose duration may vary—are structured around the program’s three core components:

1. **Individual psychosocial support**, focused on the child or adolescent, to provide emotional support, containment, and tools for comprehensive development.
2. **Family and caregiver support**, aimed at equipping families and caregivers with resources to fulfill their role as guarantors of children’s rights while also providing emotional validation and support.
3. **Coordination with social networks and significant environments**, emphasizing awareness-raising and prevention efforts to reduce discrimination and create safe, violence-free environments in key settings such as schools, health care services, and communities.

Based on these pillars and program design, the PAIG has been progressively implemented throughout the national health system since 2022.

### 4.3 Preliminary implementation data for the PAIG

Although the program began operating in 2022, only partial information regarding its implementation was accessible, specifically data covering the period from April 2023 to August 2024. The authors formally requested information from government authorities covering the first 2 years of the program’s operation, but no response had been received at the time this article was finalized. This situation points to a weakness in the program’s data registration systems—an essential component for evaluating any public policy.

Nevertheless, the available data provide a general overview of the program’s functioning, the population served, and the psychosocial services delivered.

Between April 2023 and August 2024, a total of 2,594 minors entered the program. Of these, 47 (1.8%) were children aged 3–9, and 2,547 (98.2%) were aged 10–17 years (52). The largest concentration of services (49.37%) occurred in the Central-Northern macro-region, which includes the Metropolitan Region—home to Santiago, Chile’s capital—as well as the regions of Valparaíso and Coquimbo, where approximately 56% of the country’s population resides (53).

The distribution of the intervention budget across program components is as follows:

- Services for children and adolescents: 49%
- Family guidance: 40%
- Educational inclusion initiatives: 11%

The estimated cost per service is approximately USD 90, resulting in an average cost of USD 1,264 per child or adolescent served (based on the estimated exchange rate as of March 2026). Consequently, the total investment for services provided to children, adolescents, and their families during this annual period amounted to approximately USD 2,297,480.

#### 4.3.1 Implementation milestones: political intervention, a crisis, and an opportunity

Following the publication of the Cass Report (26) in the United Kingdom, the Chilean Chamber of Deputies established a Special Investigative Commission (CEI) to gather information regarding government actions related to support plans and programs for TGNC individuals (52).

In mid-2025, the Ministry of Social Development and Family (MIDESO) convened a technical panel of experts in health and gender identity to review the design and implementation of the program. At the conclusion of their work, the panel recognized that the PAIG is essential for guaranteeing the rights of children and adolescents and recommended maintaining its current components while strengthening the program through greater standardization and the provision of stable financial resources to ensure its proper functioning (54).

Despite this positive evaluation by specialists, several media outlets and members of Congress have promoted misinformation, suggesting that the program constitutes a “gateway to hormone therapies” (55). As noted earlier, however, the PAIG does not provide any form of medical intervention; rather, it consists solely of psychosocial support.

#### 4.3.2 Training of PAIG teams

In November 2024, the Ministry of Health issued a public call for bids for the “contracting of services to deliver the initial training course for teams participating in the Gender Identity Support Program for children and adolescents (PAIG).” In April 2025, the contract was awarded to the Proyecto T team at Universidad Diego Portales. This group of professionals is part of a care program for TGNC individuals associated with the Center for Studies in Clinical Psychology and Psychotherapy at the same university.

The training course, titled “Psychosocial Support for Children and Adolescents with Diverse Gender Identities and/or Expressions,” was delivered between July and October 2025, totaling 40 instructional hours. The program was structured into four modules addressing topics such as: the development of sexuality, gender identities, and diverse sex characteristics; heteronormativity, stigma, and discrimination as explanatory frameworks for mental health problems among TGNC children and adolescents; the role of community and family as protective factors; and evidence-based approaches for working with this population. The course also examined the legal frameworks protecting children and adolescents as rights-holders in Chile and concluded with practical, case-based training focused on psychosocial support.

A total of 145 professionals from all regions of the country participated in the training, representing the psychosocial teams implementing the PAIG. Although this was the first formal training initiative offered by the Ministry of Health for PAIG professionals, the process revealed substantial differences in levels of prior knowledge and expertise among participants. Consequently, the training program provided a foundation for shared standards, conceptual coherence, and quality guidelines in the psychosocial care of TGNC children and adolescents.

#### 4.3.3 Current economic and political context

Among the various factors shaping the first 3 years of the program’s implementation, three developments are particularly noteworthy. These have emerged mainly during the past year—especially following the publication of the Cass Report in 2024—and reflect the broader social and political environment in which the PAIG has evolved.

- **Budgetary issues.**

During the annual national budget review conducted by Congress, a proposal was introduced to suspend funding for the PAIG. This proposal was driven by divergent views within Congress regarding alleged shortcomings in transparency commitments made during 2024, as well as by the absence of clear data regarding the program’s functioning and impact, specifically with hormone treatment issues. As noted earlier, the PAIG does not include hormone therapy for children or adolescents. Nevertheless, some political arguments continue to frame the program as a “gateway to hormone therapies” (55).

Chile’s Office of the Children’s Ombudsperson^4^ (56) publicly opposed the initial congressional decision to exclude PAIG funding, arguing that such a measure would represent a serious regression in the protection of children’s rights and would contradict both Chilean legislation and international human rights standards. According to this institution, the program is not optional but constitutes a legal obligation, and its suspension would result in increased vulnerability, uncertainty, and the violation of the rights of affected children and adolescents.

Following parliamentary debate in late 2025, the final decision was to maintain funding for the program at least through 2026.

- **Controversy over requests for sensitive data.**

Chile has legislation protecting privacy that specifically covers medical information, which is considered sensitive data and must be safeguarded to guarantee confidentiality and prevent misuse. Despite these protections, since late 2025, the Office of the Comptroller General of the Republic has attempted to audit the use of public resources allocated to the program by repeatedly requesting sensitive data, including the names and national identification numbers of all individuals served by the PAIG.

This request triggered public concern and a dispute between the Comptroller’s Office and organizations advocating for the protection of the privacy of minor beneficiaries. Legal actions have been filed seeking judicial protection of this data (57). At the time of writing, the matter remains under consideration by the Supreme Court of Chile.

- **Political context.**

On March 11, Chile experienced a change in presidential leadership, transitioning from an outgoing president associated with progressive left-wing politics to a newly elected president representing the conservative far right. The latter, together with several religious organizations, has opposed the idea that resolutions from the Organization of American States (OEA) and the Inter-American Commission on Human Rights should require member states, including Chile, to modify their laws in matters related to life, family, and religion (58).

Conservative and ultra-conservative sectors have mobilized around the concept of “gender ideology,” rejecting the notion of gender as a social construct (59). It stems from the Catholic Church’s resistance to the advancement of women’s rights and, in recent years, has expanded to challenge the rights of LGBTI+ people as well (60). This discourse has brought together different religious and conservative organizations by framing gender as a common threat, often without clearly defining the concept itself (59), constructing a fictional enemy that threatens biology as a strategy for moral panic (61). Scholars have described this narrative as a strategy of moral mobilization and political coalition-building (62).

Concurrently with the enactment of laws and regulations aimed at protecting gender identity in school settings, there has been a resurgence of neoconservative discourse in education aligns with neoliberal principles aimed at limiting the state’s role in matters of gender and sexuality, through which the advancement of debates on gender, sexuality, and diversity in schools has been challenged (63). Some of these movements have gained significant public visibility, such as the ‘Do not Mess with My Children’ movement, which has mobilized in the streets of the country’s capital in opposition to the LGBTQ+ rights agenda (60, 63).

This changing political environment introduces uncertainty regarding the future of the PAIG and other initiatives and legal frameworks designed to protect TGNC children and adolescents in Chile.

## 5 Discussion and recommendations

The present article aimed to describe the main characteristics, conceptual foundations, and institutional framework of the Gender Identity Support Program (*Programa de Apoyo a la Identidad de Género*, PAIG), implemented by the Chilean State since 2022 as a public policy designed to provide psychosocial support to transgender and gender non-conforming (TGNC) children and adolescents and their families. In a field characterized by clinical controversies, political debates, and structural inequalities in access to services, the analysis of the PAIG contributes empirical and conceptual insights into the possibilities and challenges involved in developing public support programs for TGNC youth within national health systems.

From a theoretical perspective, the foundations of the program align with the Minority Stress Model (17), which posits that the mental health disparities observed among sexual and gender minority populations are largely explained by sustained exposure to structural social stressors such as stigma, discrimination, expectations of rejection, and processes of internalized stigma (18, 19). As previously discussed, these factors contribute to significantly higher rates of depression, anxiety, and suicidal behaviors among TGNC individuals compared with the general population (11). Psychosocial interventions that strengthen family, community, and institutional support networks therefore function as protective factors capable of mitigating these adverse effects.

In this sense, the design of the PAIG responds to these structural determinants by providing psychological support, family guidance, and coordination with educational and community institutions. International literature has consistently shown that family support and social affirmation of gender identity are associated with better indicators of psychological well-being, higher self-esteem, and a stronger sense of personal authenticity among TGNC youth (21, 22). The explicit inclusion of families in program interventions thus constitutes a central component, as parental support has been identified as one of the most robust protective factors against the psychosocial risks associated with gender-based stigma.

Like any public policy, this program presents both strengths and weaknesses in its organization and implementation, while also facing opportunities and threats arising from the broader political and social contexts that shape national and international realities. Table 2 summarizes these elements through a SWOT^5^ analysis; however, here we highlight those aspects that were most evident during the preparation of this article.

**Table 2:** SWOT analysis of the gender identity support program (PAIG), Chile.

Strengths (internal positive factors)	Weaknesses (internal limiting factors)	Opportunities (external favorable factors)	Threats (external adverse factors)
Nationwide coverage.	Territorial heterogeneity in the availability of resources and specialized human capital.	As a state program with nationwide coverage, it represents a pioneering initiative in Latin America and globally.	National and international political and social polarization around gender identity issues, potentially affecting program continuity or design.
Comprehensive approach based on a psychosocial team addressing the emotional, social, and educational needs of children and adolescents.	Specialized training among program professionals remains insufficient and uneven and began only after program implementation.	Existence of a regulatory framework (Law No. 21.120) (47) recognizing the right to gender identity and potentially supporting the institutional consolidation of the program.	Parliamentary challenges to the program’s funding.
Active inclusion of parents, caregivers, and families as key actors in support and accompaniment processes.	Limited funding dependent on annual parliamentary budget approval, introducing structural instability.	Potential intersectoral coordination with education, child protection services, and primary health care.	Requests for access to sensitive data by oversight bodies, potentially affecting users’ trust.
Public policy addressing the well-being of a group of children and adolescents whose rights have historically been violated.	Lack of records and statistical systems providing information to evaluate program management and the implementation of operational protocols.	Generation of national evidence in public health and bioethics based on the accumulated experience of the program.	Risk of judicialization or restrictive regulatory reforms in changing political contexts.
Person-centered model that transcends an exclusively biomedical logic.	Absence of a formal national referral network and intersectoral coordination mechanisms.	Development of national systems for the evaluation and monitoring of public policies, strengthening transparency and accountability.	Public distrust that could reduce voluntary participation in the program.

SWOT, Strengths, Weaknesses, Opportunities, and Threats. NNA refers to children and adolescents.

One factor that complicates a thorough assessment of the program’s functioning—both in terms of organizational processes and outcomes—is the lack of statistical data and accessible information. It is reasonable to assume that such data exist at the local level in the various territories where the PAIG operates; however, there is currently no integrated system that would allow for broader analyses. One of the main consequences of this internal weakness is that political debates about the program’s financial viability often occur without clear and consistent data, which easily shifts the discussion toward ideological positions and structurally weakens the program’s sustainability. Avoiding ideological polarization would therefore facilitate program improvement by allowing its main strengths to be recognized.

Among these strengths, in addition to the program’s nationwide presence, it is important to highlight its strong psychosocial orientation and its focus on the lived experiences of children and adolescents. The program addresses these experiences not only at the level of individual subjectivity but also in relation to their interactions with family, educational, and broader social contexts. This comprehensive perspective—distinct from a purely biomedical model—appears better suited to addressing the needs of children and adolescents who may be at different stages of development or who experience gender incongruence in diverse ways. At the same time, the explicit inclusion of families within the intervention framework helps strengthen what is arguably the most important social support for young people’s development and well-being. Similarly, the program’s work with schools and educational institutions contributes to preventing school dropout and supports the long-term development of these children and adolescents.

Although the PAIG is formally defined as a psychosocial program—and indeed operates as such—it nevertheless shares certain elements with the health sector that, for political reasons, may not be explicitly mentioned in program technical guidelines. This refers to the possibility of referrals to specialized medical services such as gynecology, endocrinology, or urology. As with any program that operates in close connection with health services, adolescent development often requires various medical assessments. The ability of psychosocial teams to establish medical consultations, therefore, enhances the efficiency and comprehensiveness of their interventions. Moreover, intersectoral collaboration constitutes a core principle guiding health systems such as Chile’s, which require integrated work among different professional teams (64).

Consequently, medical programs addressing gender identity–related interventions need to maintain direct communication with PAIG psychosocial teams—connections that are not clearly articulated in the program’s technical guidelines (48). These discrepancies likely reflect political considerations related to the program’s institutional survival rather than genuine technical limitations in its functioning. In practice, nearly all international programs providing gender identity support (see Table 1) include at least some form of endocrinological intervention, albeit under the regulatory frameworks established in each country.

Compared with other international programs, one of the most distinctive features of the PAIG is its nationwide territorial reach. Most specialized gender identity services for TGNC youth are concentrated in university hospital centers or specialized clinics located in major cities, as is the case in the Netherlands, Sweden, or the United States (see Table 1). Even in countries where national coverage exists, services are typically centralized in a limited number of institutions. In contrast, the PAIG was designed to operate throughout the entire national territory, allowing children and adolescents and their families to access psychosocial support within their own communities.

However, this territorial decentralization also introduces important challenges for program implementation. Differences in professional training, clinical experience, and resource availability across regions may lead to variations in the quality of services provided. In countries with extensive geographic territories, such as Chile, the implementation of national programs requires robust strategies for continuous training, clinical supervision, and institutional coordination. In the Chilean context, this would entail developing training mechanisms that combine in-person and remote modalities to ensure more homogeneous standards of care.

The development of public policies addressing sexual and gender minorities is currently taking place within a climate of increasing political polarization in many countries. In some contexts, conservative political actors have promoted initiatives aimed at restricting access to gender-affirming interventions or limiting research funding in this field. These dynamics have been associated with negative effects on the psychological well-being of TGNC individuals, including increased stigma, heightened perceptions of insecurity, and additional barriers to accessing health services (38–40).

In response to these developments, several scholars have emphasized the importance of protecting and sustaining the advances achieved in public policies aimed at sexual and gender minorities. As Kennedy and colleagues (65) note, the field of health and well-being for these populations has been built through decades of academic and community collaboration, and its future depends on the ability of institutions to maintain these advances in the face of political pressures and shifting funding priorities.

Within this context, the PAIG represents a relevant example of a public policy designed to promote the well-being and rights of TGNC children and adolescents through a psychosocial, territorial, and human rights–based approach. Nevertheless, its long-term consolidation will depend on its capacity to strengthen evaluation systems, improve the training of professional teams, and further integrate the program within broader health and social protection systems.

### 5.1 Policy implications and recommendations

The descriptive analysis of the PAIG allows for the identification of several relevant implications for the development of public policies aimed at transgender and gender non-conforming (TGNC) children and adolescents.

First, it is essential to strengthen monitoring and evaluation systems for programs of this type by the creation of integrated databases that enable the analysis of coverage, care trajectories, and outcomes in psychological and social well-being. The availability of robust empirical evidence would not only improve the quality of interventions but also help sustain such programs in the face of political debates and ideological challenges.

Second, systematic strategies for training and clinical supervision should be developed for the professional teams responsible for implementing these programs across different territories. Regional heterogeneity in professional capacities can be reduced through continuous training initiatives, interdisciplinary supervision networks, and spaces for the exchange of best practices among teams.

Third, it is necessary to strengthen coordination between psychosocial support services and specialized medical services, ensuring that young people who require medical assessments or clinical interventions can access them within ethical, prudent, and evidence-based frameworks. Such coordination is consistent with international models of comprehensive care for TGNC youth (14).

Fourth, public policies should prioritize support for families and communities as a central component of interventions. International evidence shows that parental support, school inclusion, and a sense of community belonging constitute fundamental protective factors for the psychological well-being of TGNC youth.

Finally, in the context of growing political polarization surrounding gender identities in many countries, it is crucial to consolidate institutional frameworks grounded in human rights that guarantee access to psychosocial support and health services for TGNC children and adolescents. Protecting the well-being of this population requires sustained public policies, rigorous scientific evidence, and ongoing collaboration among academic institutions, health systems, and community organizations.

This study presents several limitations that should be considered when interpreting its findings. First, the analysis is primarily based on the institutional description of the program and publicly available documentation,^6^ which limits the ability to empirically evaluate the outcomes of interventions in terms of psychological well-being or developmental trajectories among participants. Second, the absence of nationally systematized data on the functioning of the PAIG limits the capacity to analyze territorial variations in the program’s implementation. Future research could benefit from incorporating both qualitative and quantitative methodologies to better understand the experiences of young participants, their families, and the professionals involved, thereby generating knowledge that may contribute to improving the program’s implementation.

Nevertheless, this article illustrates how the PAIG represents an innovative initiative within the current international landscape of public policies directed toward TGNC youth, particularly due to its psychosocial approach and nationwide territorial reach. Although the program faces challenges related to implementation heterogeneity and the need to strengthen its evaluation systems, it represents a significant step forward in the institutional recognition of the support needs of children and adolescents experiencing gender incongruence.

In conclusion, in a global context characterized by intense debates regarding the care of TGNC youth, the development of programs such as the PAIG underscores the importance of sustaining evidence-based public policies oriented toward well-being and grounded in human rights principles. Strengthening evaluation systems, consolidating professional training, and promoting research on program outcomes will be essential for ensuring the program’s continuity and for contributing to the development of support models capable of responding ethically and effectively to the needs of this population.

## Footnotes

1 The authors acknowledge the wide diversity of sexual and gender identities that exist. However, in this article we use the acronym TGNC to refer to transgender and gender non-conforming individuals. This term commonly includes children and adolescents who experience discomfort with the gender assigned to them at birth, but who may not yet identify themselves as transgender.

2 Gender dysphoria is the term used in DSM-V-TR (76) for referring adolescents and adults’ individuals who expressed a marked incongruence between one’s experienced/expressed gender and assigned sex at birth, causing significant distress or impairment in functioning, lasting at least 6 months. For the case of children, the name is Gender Dysphoria in Children and separated the diagnosis from that of gender dysphoria in adolescents and adults and set more stringent behavioral criteria. In all cases, the diagnosis focuses on the severe distress rather than on the identity. On the other hand, Gender Incongruence is the term used in ICD-11 (13) to refer to the children’s experience of having a gender that does not match the sex you were assigned at birth. This last term is used more frequently in the article because it is the terminology used by the Chilean Ministry of Health in the PAIG program.

3 At present, only one non-governmental organization, Juntos Contigo, is officially accredited to provide services under this framework. According to information published on its website, more than 600 families have received support from this institution to date. However, the Ministry of Health (MINSAL) does not currently maintain statistical records or a monitoring system that reports on the functioning or outcomes of this organization.

4 The Office of the Children’s Ombudsperson in Chile is an autonomous public legal institution established. Its mandate is to promote, protect, and disseminate the human rights of children and adolescents. The institution operates independently from the government and oversees the fulfillment of children’s rights by both the State and society.

5 A qualitative descriptive approach was used to conduct the SWOT analysis. Relevant information was obtained through a documentary review of national regulations, official program reports, and peer-reviewed literature addressing gender identity support policies in Chile. Data were analyzed using thematic content analysis to identify key factors, which were subsequently categorized into strengths, weaknesses, opportunities, and threats, distinguishing between internal and external elements. The process was conducted iteratively by the authors until consensus was reached. Given the interpretative nature of SWOT analysis, the findings are intended to provide a structured synthesis rather than causal inference.

6 The research team submitted a request to the Health Ministry on November 24, 2025, under the Transparency Law to obtain information regarding the number of individuals who have benefited from the PAIG since its implementation. However, at the time of writing, this request remains unanswered.
